# Inhibition of miR-490-5p Promotes Human Adipose-Derived Stem Cells Chondrogenesis and Protects Chondrocytes via the PITPNM1/PI3K/AKT Axis

**DOI:** 10.3389/fcell.2020.573221

**Published:** 2020-11-09

**Authors:** Hongyi Li, Xiaoyi Zhao, Xingzhao Wen, Anyu Zeng, Guping Mao, Ruifu Lin, Shu Hu, Weiming Liao, Zhiqi Zhang

**Affiliations:** ^1^Department of Joint Surgery, The First Affiliated Hospital of Sun Yat-sen University, Guangzhou, China; ^2^Guangdong Provincial Key Laboratory of Orthopedics and Traumatology, Guangzhou, China

**Keywords:** chondrocytes, human adipose-derived stem cells, miRNA-490-5p, osteoarthritis, PI3K/AKT

## Abstract

MicroRNAs (miRNAs) play a pivotal role in cartilage development and homeostasis in osteoarthritis (OA). While the fundamental roles of miRNAs in cartilage degeneration have been extensively studied, their effects on chondrogenic differentiation induced by human adipose-derived stem cells (hADSCs) and the underlying mechanisms remain largely elusive. Here, we investigated the roles and mechanisms of miRNAs in hADSC chondrogenic differentiation and chondrocyte homeostasis. Using microarray analysis, we screened miRNAs expressed in the chondrogenic differentiated hADSCs and identified miR-490-5p as the most significantly down-regulated miRNA. We analyzed its expression patterns during chondrogenesis *in vivo* and *in vitro*. Our study showed that miR-490-5p overexpression promoted the transition of hADSCs from chondrogenesis to osteogenesis. In addition, based on miRNA–mRNA prediction analysis and dual-luciferase reporter assay, we proposed and proved that miR-490-5p targeted PITPNM1 by binding to its 3′-UTR and inhibiting its translation. Moreover, loss- and gain-of-function experiments identified the involvement of the PI3K/AKT signaling pathway, and a rescue experiment determined the effect and specific mechanism of the miR-490-5p/PITPNM1/PI3K/AKT axis in hADSC chondrogenic differentiation and chondrocyte homeostasis. Inhibition of miR-490-5p alleviated cartilage injury *in vivo* as demonstrated using the destabilization of the medial meniscus (DMM) OA model. We identified miR-490-5p as a novel modulator of hADSC-mediated chondrogenesis and chondrocyte phenotype. This study highlighted that miR-490-5p attenuated hADSC chondrogenesis and accelerated cartilage degradation through activation of the PI3K/AKT signaling pathway by targeting PITPNM1. Inhibition of miR-490-5p facilitated hADSC chondrogenic differentiation and protected chondrocyte phenotype via the PITPNM1/PI3K/AKT axis, thus providing a novel stem cell potential therapeutic target for OA treatment.

## Introduction

The prevalence of osteoarthritis (OA), a degenerative disease, is increasing because of aging and increasing obesity in the global population (Hunter and Bierma-Zeinstra, 2019). However, its exact pathogenesis remains unclear and has led to the lack of effective interventions to stop the progression of OA. Studies have suggested that irreversible cartilage degeneration might be the key physiopathologic basis for OA (Li et al., 2017). However, overwhelming evidence indicates that chondrogenesis induced by bone mesenchymal stem cells (MSCs) could be a promising therapeutic target for the treatment of OA (De Bari and Roelofs, 2018). Recently, several studies have suggested that compared with MSCs, human adipose-derived stem cells (hADSCs) are more accessible as well as have great multilineage potential, thus promoting hADSCs as an ideal seeding cell type for tissue engineering and regenerative therapies (Naderi et al., 2017; Liu et al., 2019).

MicroRNAs (miRNAs, < 22 bp) are considered to play vital roles in post-translational regulation of gene expression by sponging messenger RNAs (mRNAs); their impaired function is closely related to many complex diseases including OA (Coutinho de Almeida et al., 2019). miRNAs are associated with epigenetic regulatory mechanisms of proliferation, migration, or apoptosis in chondrocytes, such as DNA methylation (Sun et al., 2018), acetylization (Meng et al., 2018), phosphorylation (Golden et al., 2017), and ubiquitination (Li et al., 2014). Thus, miRNA-regulated chondrogenesis in hADSCs opens promising avenues for the development of targeted therapies for OA.

According to available evidence, the PI3K/AKT/mTOR signaling pathway that is essential for normal metabolism of joint tissues is also involved in the development of OA. PITPNM1 (NIR2) gene encodes the human phosphatidylinositol transfer membrane protein (Ocaka et al., 2005). It is a member of a protein family that shares homology with the Drosophila retinal degeneration B protein (Litvak et al., 2002) and functions as a phosphatidylinositol-transfer protein (Kim et al., 2013). However, the exact effect on hADSC chondrogenesis and chondrocyte phenotype as well as the mechanism of miR-490-5p/PITPNM1/PI3K/AKT have not been elucidated.

We have previously analyzed the miRNA expression profile of hADSCs during chondrogenesis using miRNA microarray and identified the differentially expressed miRNAs (Zhang et al., 2012). Subsequently, the up-regulated miRNAs were found to be closely associated with the regulation of cartilage development, homeostasis, and degeneration (Hu et al., 2019; Sun et al., 2019). However, the effect and molecular mechanisms involved in the interaction of down-regulated miRNAs, such as miR-490-5p, with chondrogenesis in hADSCs have not yet been fully elucidated. Hence, the present study aimed to investigate how miR-490-5p affects chondrogenesis in hADSCs with respect to the PITPNM1/PI3K/AKT axis in order to find a novel therapeutic target for OA.

## Materials and Methods

### Patient Recruitment, Sample Collection, Cell Culture, and Chondrogenic Induction

This study was approved by the First Affiliated Hospital of Sun Yat-sen University Clinical Research Ethics Committee ([2013] C-110). All participants in this study provided signed informed consents. Adipose specimens were acquired from healthy young adults who underwent liposuction (age < 30 years) at the First Affiliated Hospital of Sun Yat-sen University. hADSCs were separated and cultured by collagenase digestion and related procedures as previously described (Bunnell et al., 2008). Based on a previously described protocol (Zhang et al., 2012), chondrogenesis was induced in hADSCs of passage 3 using the microsphere culture technique. Briefly, hADSCs were cultured in incomplete chondrogenic medium (Cyagen Biosciences, China) with or without TGF-β3 in a 24-well plate for 3, 7, 14, 21, 28, or 35 days, respectively (Figure 2A).

Degenerated cartilage specimens of knee or hip joints were acquired from 18 patients (5 males, 13 females), with an average age of 74 years, during arthroplasty surgeries. Normal cartilage specimens were acquired from 16 patients (nine males, seven females), with an average age of 61.4 years, who did not have OA or rheumatoid arthritis and underwent hip arthroplasty surgery for fractures of the femoral neck; these were considered as controls. Chondrocytes from six pairs of OA and control specimens were isolated and cultured as previously described (Dai et al., 2020). Further, RNA from 12 pairs of OA and control specimens was extracted, reverse transcribed, and used for qRT-PCR as described previously (Dudek et al., 2017). GAPDH was used as the internal reference gene for mRNA expression evaluation, while U6 was applied as the internal reference gene for miRNA expression evaluation.

Twenty-six patients with OA (20 females and 6 males) and corresponding healthy control participants (15 females and 10 males) were enrolled for analyzing circulating miRNA biomarkers in plasma by qRT-PCR. The average age of the enrolled patients with OA and healthy participants was 68.4 and 54.6 years, respectively. The enrollment criteria depended on typical clinical history, symptoms of pain, signs of swelling or limitation, and X-ray imaging (Kellgren-Lawrence grade ≥ 2) (Kohn et al., 2016). The exclusion criteria have been added as follows: patients with rheumatoid arthritis or other inflammatory arthritis, and patients who were taking medications such as corticosteroid that may influence cartilage metabolism. The procedures for sample collection and examination were carried out as described in previous studies (Kroh et al., 2010; Meng et al., 2018). Cel-miR-39 was used as the spiked-in control. The primers for qRT-PCR were shown in Supplementary Table 1.

### miRNA Microarray Analysis

**FIGURE 1:**
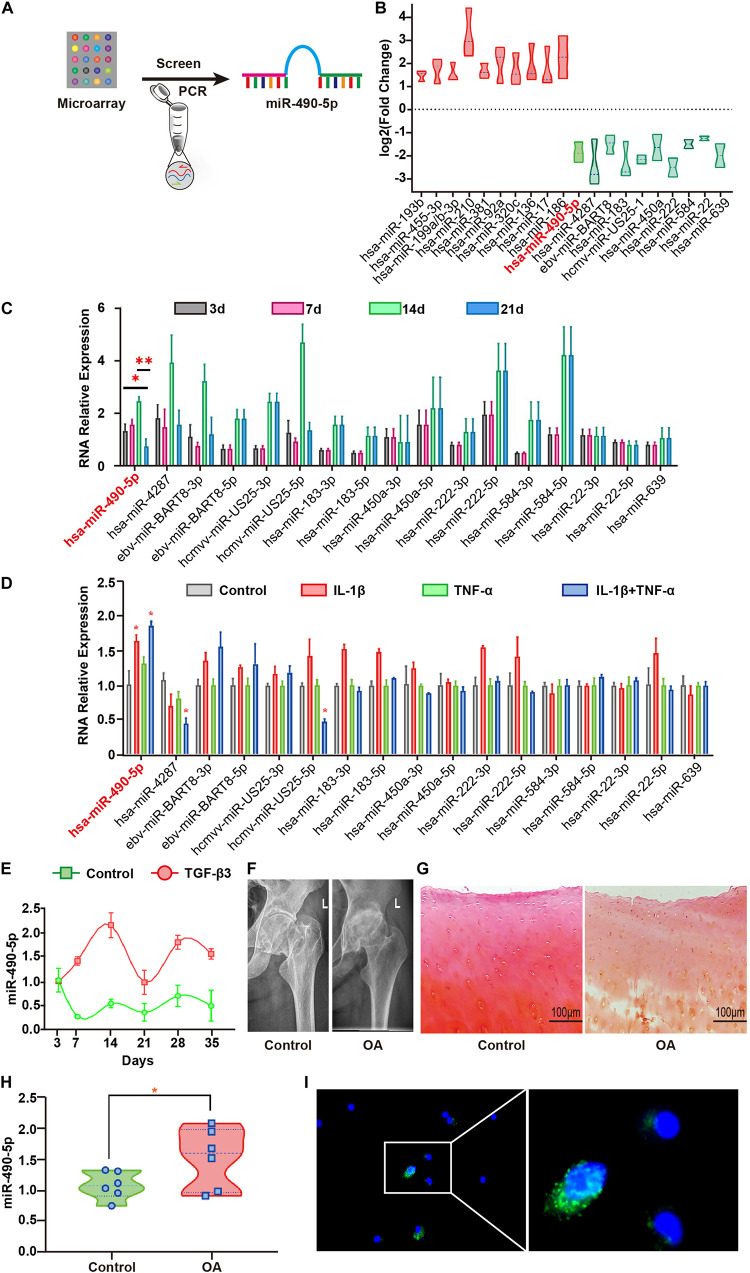
Hsa-miR-490-5p was elevated during the middle stage of hADSC chondrogenic differentiation and in OA. **(A)** Flowchart depicting screening of hsa-miR-490-5p. **(B)** miRNA microarray revealed the 10 most significant miRNAs showing differential expression during hADSC chondrogenesis; red: up-regulated; green: down-regulated. **(C)** Quantitative real time PCR (qRT-PCR) validated the expression level of the down-regulated miRNAs during chondrogenic differentiation of hADSCs on days 3, 7, 14, and 21. **(D)** The expression of chondrogenesis down-regulated miRNAs in the human chondrocytes stimulated with either IL-1β (5 ng/ml) or TNF-α (10 ng/ml) or both for 24 h. **(E)** Gene expression levels of miR-490-5p, evaluated by qRT-PCR, in hADSCs that were induced to undergo chondrogenesis with or without TGF-β3 for 3, 7, 14, 21, 28, and 35 days, respectively. **(F)** X-ray imaging showing the hip joints of patients with OA and controls. **(G)** Safranin-O/fast green staining of the cartilage from patients with OA and controls; scale bar: 100 μm. **(H)** Gene expression levels of miR-490-5p, evaluated by qRT-PCR, in chondrocytes from patients with OA (*n* = 6) and controls (*n* = 6). **(I)** RNA FISH indicated that the predominant location of miR-490-5p was in the cytoplasm. MiR-490-5p probes were labeled with FAM (green). Nuclei were stained with DAPI. Scale bar: 10 μm. *p < 0.05, **p < 0.01, n.s.: no significance.

Three pairs of hADSCs samples before and after chondrogenic differentiation were used for microarray analysis using miRCURYTM LNA Array, 5th generation (v.14.0) (Exiqon, Vedbæk, Denmark), which contains more than 1891 capture probes, covering all human, mouse, and rat miRNAs annotated in miRBase 14.0. Microarray analysis was performed according to the manufacturer’s protocol and as described in our previous study (Zhang et al., 2012). The 10 most significant differently down-regulated miRNAs were further analyzed in the present study (Figure 1A).

### Characterization of hADSCs and Flow Cytometry Analysis

Directional differentiation mediums (human MSC differentiation medium for osteogenesis, adipogenesis, or chondrogenesis, respectively, Cyagen, China) induced hADSCs to undergo osteogenic, adipogenic, or chondrogenic differentiation, and Alizarin Red, Oil Red O, and Alcian Blue stains were used to identify the differentiated hADSCs, respectively. Flow cytometry was used to identify the surface antigens of hADSCs (CD29, CD44, CD59, CD105), fibroblast (HLA-DR), and hematopoietic (CD34, CD45) cell lines. Monoclonal antibodies (BioLegend, Inc., San Diego, CA, United States) for the abovementioned antigens were used for detection. Mouse/Rabbit IgG monoclonal antibody (BioLegend, Inc., San Diego, CA, United States) was used as a negative control.

### Assay to Detect the Proliferation Abilities of Chondrocytes

The effect of knockdown or overexpression of miRNA on the proliferation of PHCs was studied using the Cell Counting Kit-8 (CCK-8; Sigma-Aldrich, United States) as described previously (Xu et al., 2019).

### Wound Healing Assay

A wound healing assay was used to examine the influence of miRNAs on the migration of chondrocytes by observing images in the same position before and after treatment as described previously (Liang et al., 2007). Briefly, chondrocytes were seeded into six-well plates and cultured until they reached confluency. The 100-μl pipette tip was used to make a straight scratch to simulate a wound. DMEM/F12 medium without fetal bovine serum was used for cell culture 4 h before scratch and at all times after scratch. The images were acquired using a fluorescence microscope (NIKON Eclipse Ti-E, Japan) at 0, 24, and 48 h, respectively. The size of the wounds was measured by ImageJ software (National Institutes of Health, United States).

### Transfection

The hADSCs and PHCs were transfected with miR-490-5p mimic or inhibitor (RiboBio, Guangzhou, China) at a concentration of 50 or 100 nM, and PITPNM1 siRNA or negative control (Ribo Bio) using Lipofectamine^®^ 3000 Transfection Reagent (Gibco Life Technologies, United States) according to the manufacturer’s instructions. Cells were collected after 48 h for qRT-PCR analysis and after 72 h for WB analysis. Before inducing chondrogenesis in hADSC microsphere cultures, hADSC monolayers at passage 3 were transfected on the day after plating and 3 days after plating, respectively, as a prior study described (Mao et al., 2018). The sequences were shown as follows: miR-490-5p mimic: 5′-CCAUGGAUCUCCAGGUGGGU-3′, 5′-ACCACCUGCGA GAUCCAUGG-3′; miR-490-5p inhibitor: 5′-ACCCACCUGGA GAUCCAUGG-3′; miR-490-5p antagomir: 5′-ACCCACCUGG AGAUCCAUGG-3′; siPITPNM1: CGAGGCAGCTAAAGGC ATT. And the RiboBio NCs were used as their negative controls respectively. All of the reagents were purchased from RiboBio (Guangzhou, China).

### Western Blot Analysis

WB analysis was performed as per the standard protocol. Primary antibodies against COL2A1 (1:1000, #ab188570, Abcam), AGGRECAN (1:100, #ab3778, Abcam), SOX9 (1:1000, #ab185230, Abcam), RUNX2 (1:1000, #ab76956, Abcam), MMP13 (1:3000, #ab39012, Abcam), ADAMTS5 (1:1000, #ab41037, Abcam), ALP (1:500, #ab83259, Abcam), CEBPα (1:500, #ab40764, Abcam), PPAR**γ** (1:500, #ab45036, Abcam), PITPNM1 (NIR2, 1:1000, #ab254959, Abcam), AKT (1:10000, #ab8805, Abcam), pAKT (1:000, #ab8933, Abcam), PTEN (1:2000, #ab170941, Abcam), GSK-3β (1:5000, #ab32391, Abcam), mTOR (1:1000, #ab109268, Abcam), and GAPDH (1:2000, #2118, CST) were used. The protein bands were detected by ChemiDoc Touch (BIO-RAD, United States) and analyzed using Image Lab^TM^ (BIO-RAD, United States). The intensity of bands was compared by Image J software. *In situ* hybridization and IHC analysis were performed with the corresponding miRNA probes (RiboBio, Guangzhou, China) and the abovementioned antibody as described previously (Mao et al., 2018).

### Immunofluorescence (IF)

IF analysis of the transfected cells (after 48 h) was performed as described previously (Yamasaki et al., 2016). The primary antibody was the abovementioned antibody, and the secondary antibodies were conjugated to goat anti-rabbit IgG (Alexa Fluor^®^ 555, #4413S, Cell Signaling Technology, United States). Images were captured with a confocal laser microscope (LSM 780, Zeiss, Germany) with a 40 × objective.

### RNA Fluorescence *in situ* Hybridization (FISH)

The cells were cultured on glass coverslips. Fixed cells were permeabilized with 0.1% Triton X-100 and 10 mM VRC CSK buffer and treated with RNase R at 37°C for 15 min and then fixed again. The fixed cells were dehydrated by 70%, 80%, and 100% ethanol, respectively. Then, hybridization was performed at 37°C overnight. The slices were washed three times by 4, 2, and 1 × SSC buffer, respectively. Then, the slices were stained with 2 μg/ml DAPI for 10 min at room temperature. The images were acquired using a confocal microscopy (Zeiss LSM780, Germany). Fluorescence intensity was analyzed by ImageJ. The fluorescent oligonucleotide probe for miR-490-5p was synthesized from Servicebio (Wuhan, China). The probe sequences were shown as below: miR-490-5p: 5′-biotin labeling-ACCCACCTGGAGATCCATGG-3′ (two-tailed FAM).

### Dual-Luciferase Reporter Assay

The dual-luciferase reporter assays were performed as per the protocol. Briefly, SW1353 cells (human chondrosarcoma cell line) were co-transfected with the indicated RNA oligonucleotides and the reporter plasmids. After incubation for 24 h, SW1353 was transfected with a miRNA mimic or inhibitor. Luciferase activities were determined after 48 h using the Dual-Luciferase Reporter Assay System (E1910, Promega, United States).

### Destabilization of the Medial Meniscus (DMM) Model of OA

The animal experiments in the present study adhered to institutional review board-approved protocols, approved by ICE for animal research of the First Affiliated Hospital of Sun Yat-sen University ([2013]A-110). Male wild-type (WT) C57 BL/6 J (purchased from GemPharmatech, Co., Jiangsu, China) were housed under specific pathogen-free conditions and used in experiments at 10 weeks of age. The mice were provided with a normal diet and had access to water *ad libitum*. The mice were anesthetized by isoflurane inhalation for operation. The mice were exposed to 2–3% isoflurane for anesthesia induction, and then 1.5–2% isoflurane for anesthesia maintenance. The humane endpoint in this *in vivo* study was the inability to rise or ambulate; if a mouse suffered severe OA and was correlated with the inability to access food or water, the mouse would be euthanized. Briefly, to induce OA *in vivo*, the mice were subjected to DMM surgery of the right knees. Their left knees were sham operated as control. After 4 weeks, all mice were randomly divided into four cohorts (*n* = 8/cohort): sham, DMM, antagomir-control, and antagomir-490-5p. Mice from DMM, antagomir-control, and antagomir-490-5p cohorts were administered with multiple intra-articular injections of 10 μl of saline, antagomir-control, or antagomir-490-5p for 4 weeks (once a week), respectively. After 8 weeks, the mice were sacrificed and the knee joints were harvested from these mice and further analyzed by qRT-PCR and IHC.

### Statistical Analyses

All experiments were performed with three biological replicates. Data are expressed as mean ± standard deviations (SD). The independent *t*-test and Mann–Whitney *U*-test were used to identify differences between cohorts. One-way analysis of variance (ANOVA) and Kruskal–Wallis tests were carried out for multiple group comparisons. Data analyses were performed using SPSS Version 20 (IBM Corporation, Armonk, NY, United States). Statistical significance was considered at *p* < 0.05.

## Results

### Expression Pattern of miR-490-5p During Chondrogenic Differentiation of hADSCs

Microarray analysis of three pairs of undifferentiated versus differentiated samples identified 312 up-regulated and 252 down-regulated miRNAs (Supplementary Table 2). To better understand the roles of down-regulated miRNAs in chondrogenesis, we first determined the expression pattern of the 10 miRNAs that showed the most significant differential down-regulation in hADSCs induced to undergo chondrogenesis (Figure 1B). The expression level of miR-490-5p in chondrogenic differentiated hADSCs on day 21 was significantly lower among the 10 down-regulated miRNAs as compared to that on days 3, 7, and 14 (Figure 1C). Therefore, miR-490-5p was selected for further analyses. To examine the expression pattern of the miR-490-5p *in vitro* OA model, primary human chondrocytes (PHCs) were subjected to 5 ng/ml interleukin (IL)-1β and 10 ng/ml tumor necrosis factor (TNF)-α; qRT-PCR analyses showed up-regulated expression of miR-490-5p (Figure 1D). miR-490-5p expression was further validated in the whole process of chondrogenesis. It was elevated in hADSCs induced to undergo chondrogenic differentiation on days 7–14, dropped significantly on day 21, showed a slight rebound on day 28, and finally a mild decline on day 35 (Figure 1E). Moreover, the expression of miR-490-5p was evaluated in three pairs of cartilage tissues obtained from OA and control (Figures 1F,G), and the up-regulated expression of miR-490-5p was determined in chondrocytes isolated from OA cartilage by qRT-PCR (Figure 1H). RNA FISH showed that the expression of miR-490-5p in cytoplasm was more than that in nucleus of PHCs; that is, miR-490-5p was mainly localized in the cytoplasm (Figure 1I).

### miR-490-5p Attenuated the Chondrogenesis of hADSCs

**FIGURE 2:**
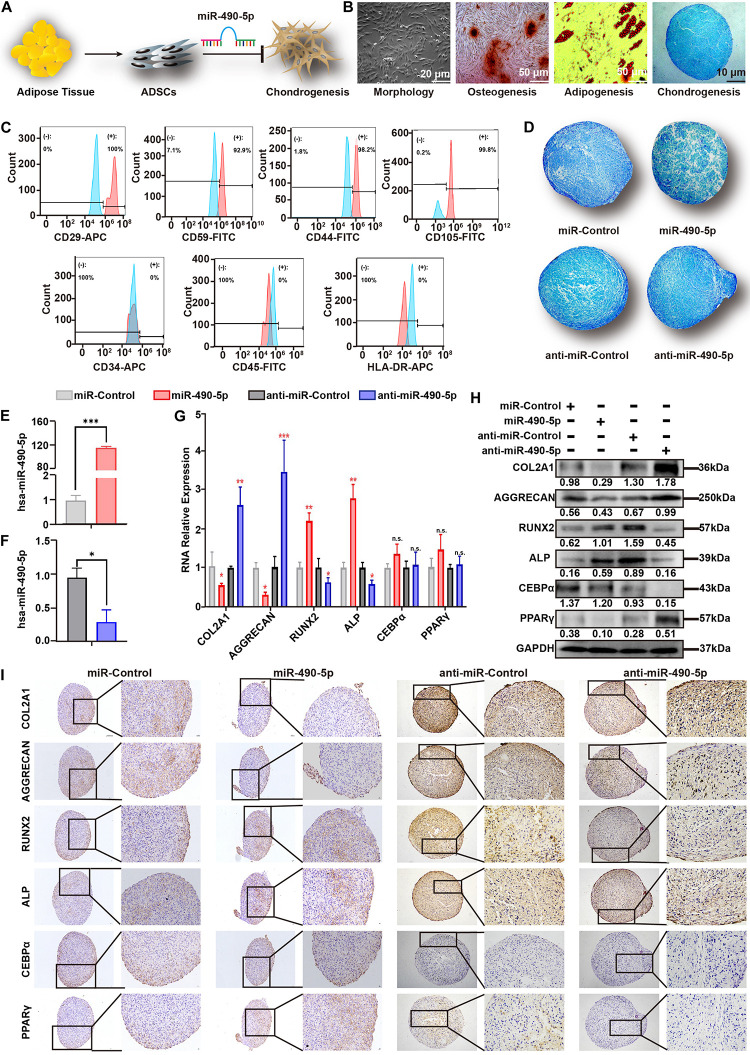
miR-490-5p attenuated hADSC chondrogenesis. **(A)** Flowchart depicting hADSC isolation and impact on chondrogenesis. **(B)** hADSCs presented a typical morphology of spindle shake; the multilineage potential for osteogenesis, adipogenesis, and chondrogenesis was shown. **(C)** hADSCs were identified by flow cytometry; mesenchymal positive markers included CD29, CD59, C44, and CD105, and negative markers included CD34, CD45, and HLA-DR; light blue histograms denoted the isotype controls, and the light red peak denoted the tested markers. hADSCs were transfected with miR-490-5p or anti-miR-490-5p and then induced to undergo chondrogenesis in 3D culture for 14 days. **(D)** Alcian Blue staining was performed in the pathological section of microspheres. The efficiency of the miR-490-5p mimic or inhibitor was qualified **(E,F)**. **(G)** Gene expression of selected chondrogenic markers (COL2A1 and AGGRECAN), osteogenic markers (ALP and Runx2), and adipogenic markers (CEBPα and PPARγ) was evaluated by qRT-PCR in cells from the above microspheres. Protein expression of COL2A1, AGGRECAN, ALP, RUNX2, CEBPα, and PPARγ from the above microspheres was studied by **(H)** WB and **(I)** IHC. *p < 0.05, **p < 0.01, ***p < 0.001, n.s.: no significance.

The hADSCs of passage 3 (P3) were identified by their typical morphology, ability for pluripotent differentiation, and flow cytometry. Figure 2B showed the typical hADSC morphology with isolated spindle cells. After culturing P3 hADSCs in specific inducing medium for 21 days, the pluripotent differentiation potential was determined by staining with Alizarin Red (osteogenesis), Oil Red O (adipogenesis), and Alcian Blue (chondrogenesis) (Figure 2B). Subsequently, flow cytometer demonstrated that these cells were positive for stem cell markers (CD29, CD44, CD59, and CD105) and negative for HLA-DR (exclusion of fibroblast), CD45, and CD34 markers (exclusion of hematopoietic cell lines) (Figure 2C). This indicated the successful isolation of hADSCs with pluripotent differentiation potential.

To further investigate the role of miR-490-5p in chondrogenesis, miR-control, miR-490-5p, anti-miR-control, and anti-miR-490-5p were transfected into hADSCs, respectively. Then, the hADSCs of the above four groups were induced to differentiate into the chondrogenic microspheres for 21 days. Alcian Blue staining revealed that overexpression of miR-490-5p attenuated the chondrogenesis of hADSCs, while its knockdown promoted chondrogenesis (Figure 2D). The transfection efficiency of the miR-490-5p mimic or inhibitor was qualified (Figures 2E,F). In order to comprehensively understand the effect of miR-490-5p on chondrogenesis, osteogenesis, and adipogenesis, the gene and protein expression of selected chondrogenic markers (COL2A1 and AGGRECAN), osteogenic markers (ALP and RUNX2), and adipogenic markers (CEBPα and PPARγ) were assessed in the four groups of microspheres by qRT-PCR (Figure 2G), Western blotting (WB) (Figure 2H), and immunohistochemistry (IHC, Figure 2I); overexpression of miR-490-5p was found to decrease the expression of COL2A1 and AGGRECAN, while its knockdown increased the expression of chondrogenic markers. In stark contrast, the expression patterns of ALP and RUNX2 in the four groups of microspheres were opposite to that of the chondrogenic markers. There was no significant change in the expression of CEBPα and PPARγ among the four groups of microspheres. The abovementioned results suggested that miR-490-5p could inhibit the chondrogenic potential and facilitate the osteogenic potential of hADSCs; however, the adipogenic potential of hADSCs was not significantly affected by miR-490-5p.

### miR-490-5p Impaired the Chondrocyte Proliferation and Migration Abilities

**FIGURE 3:**
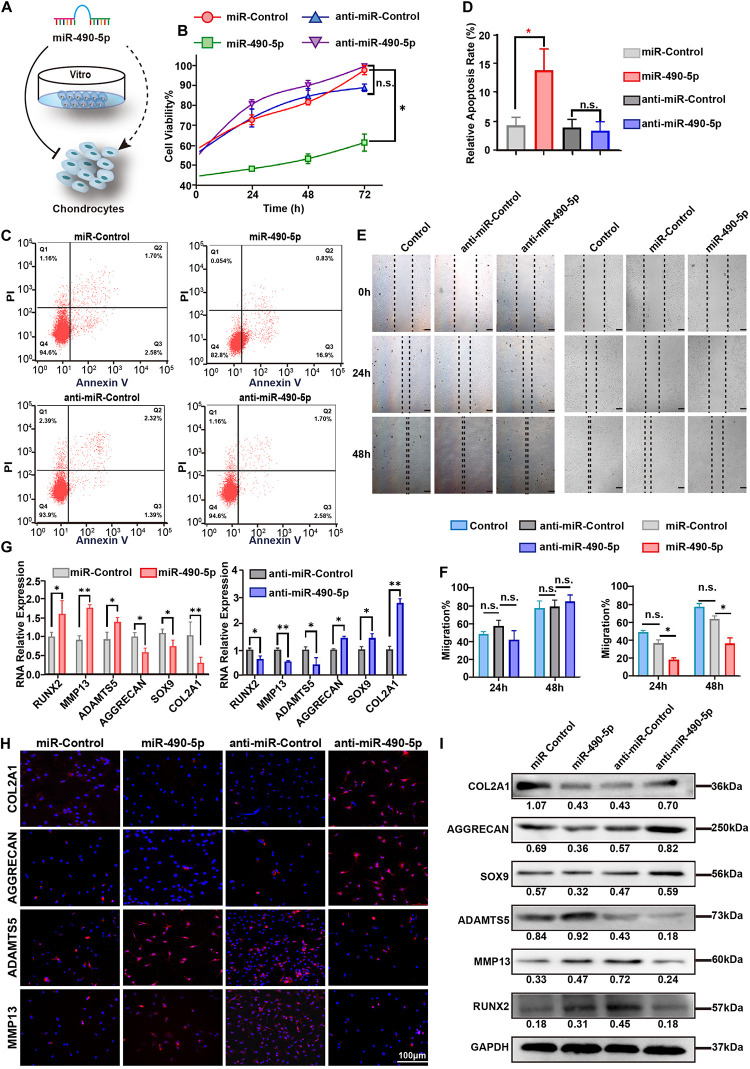
miR-490-5p had a negative impact on proliferation and migration of chondrocytes. **(A)** Diagrammatic representation of the effect of miR-490-5p on chondrocytes *in vitro*. **(B)** Primary human chondrocytes (PHCs) were transfected with miR-490-5p or anti-miR-490-5p, and CCK8 showed the proliferation ability of transfected PHCs for 24, 48, and 72 h. **(C,D)** Apoptosis was evaluated by flow cytometry after 48 h. **(E,F)** Scratch wound assays were performed to test the migration ability of PHCs transfected with anti-miR-490-5p for 24 and 48 h. Expression levels of extracellular matrix anabolic and catabolic markers including COL2A1, AGGRECAN, SOX9, MMP13, ADAMTS5, and RUNX2 from transfected PHCs were detected by qRT-PCR **(G)**, immunofluorescence **(H)**, and Western blot **(I)**. ^∗^*p* < 0.05, **p < 0.01, n.s.: no significance.

To characterize the effect of miR-490-5p on chondrocytes, miR-490-5p was either knocked down or overexpressed in chondrocytes (Figure 3A). First, the cell viability and apoptosis rate of the chondrocytes were evaluated by CCK-8 assay and flow cytometry, respectively. Overexpression of miR-490-5p inhibited chondrocyte proliferation and increased the rate of chondrocyte apoptosis significantly (*p* = 0.0321 and *p* = 0.0025, respectively); however, miR-490-5p knockdown did not markedly affect the cell viability or the apoptosis rate of chondrocytes (*p* = 0.6874 and *p* = 0.6555, respectively) (Figures 3B–D). Further, wound healing assay showed that the overexpression of miR-490-5p significantly suppressed the motility of chondrocytes while knockdown of miR-490-5p could not promote the motility of chondrocytes significantly (Figures 3E,F). Subsequently, as indicated by qRT-PCR analysis (Figure 3G), the knockdown of miR-490-5p resulted in down-regulated expression of extracellular matrix (ECM) catabolism markers (MMP13, RUNX2, and ADAMTS5) and up-regulated expression of ECM anabolism markers (COL2A1, AGGRECAN, and SOX9). On the contrary, the overexpression of miR-490-5p caused reversal in the expression patterns of ECM catabolism and anabolism markers. The same trends were observed in both IF (Figure 3H) and WB (Figure 3I) analyses. These above findings suggested that miR-490-5p not only weakened the proliferation and migration abilities of chondrocytes but also facilitated the catabolism of ECM in chondrocytes.

### Identification and Analysis of PITPNM1-Related miR-490-5p Sponge in PHCs

**FIGURE 4:**
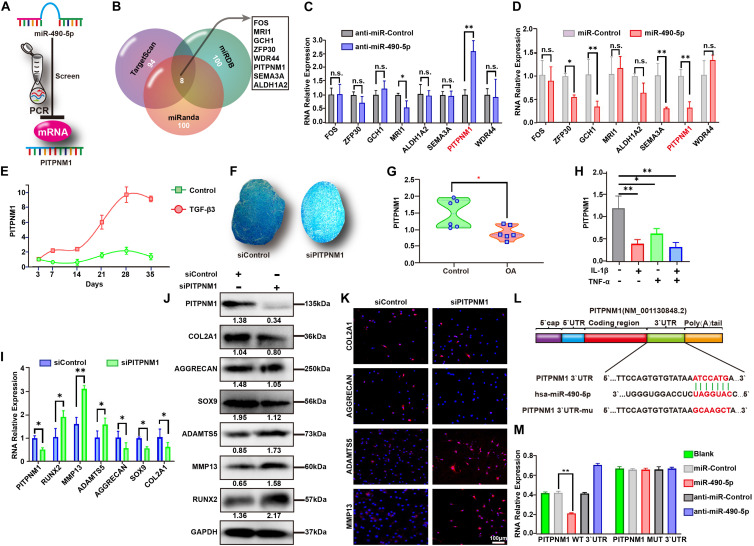
PITPNM1 was screened and identified as a sponge for miR-490-5p in PHCs. **(A)** Schematic illustration showing the flowchart for screening of PITPNM1 targeted by hsa-miR-490-5p. **(B)** Sketch showing the overlapping of the predicted miR-490-5p target mRNAs among TargetScan, miRDB, and miRanda database. Gene expression of the eight overlapped target mRNAs was validated by qRT-PCR in PHCs transfected with **(C)** anti-miR-490-5p and **(D)** miR-490-5p. **(E)** Gene expression levels of PITPNM1 were evaluated by qRT-PCR in hADSCs that were induced to undergo chondrogenesis with or without TGF-β3 for 3, 7, 14, 21, 28, and 35 days. **(F)** After PITPNM1 was knocked down, hADSCs were induced to undergo chondrogenesis in 3D culture for 14 days; Alcian Blue was used to stain pathological section of microspheres. **(G)** Gene expression of PITPNM1 in chondrocytes from patients with OA and controls was determined by qRT-PCR (*n* = 6). **(H)** The expression of PITPNM1 in PHCs stimulated with either IL-1β (5 ng/ml) or TNF-α (10 ng/ml), or both for 24 h. After PITPNM1 was knocked down in PHCs, the expression levels of COL2A1, AGGRECAN, SOX9, MMP13, ADAMTS5, and RUNX2 were examined by **(I)** qRT-PCR, **(J)** Western blot, and **(K)** immunofluorescence. **(L)** Schematic illustration showing the flowchart of mutant predicted binding site in PITPNM1 for hsa-miR-490-5p. **(M)** The relative luciferase activity was tested by the Dual-Luciferase Reporter System. *p < 0.05, **p < 0.01, n.s.: no significance.

To further identify the downstream mRNA target of miR-490-5p (Figure 4A), a Venn diagram was used to analyze 95 predicted miR-490-5p targets obtained from TargetScan (Cumulative weighted context++ score ≤ −0.4), the targets with top 100 binding stability from miRanda (mirSVR score ≤ −1.18), and those with the top 100 target scores form miRDB (Supplementary Table 3). Eight predicted targets were found to overlap among the three databases (Figure 4B), and among these, PITPNM1 was found to be the strongest miR-490-5p-associated target as determined by qRT-PCR (Figures 4C,D). Furthermore, the expression of PITPNM1 in hADSCs during chondrogenic differentiation was found to follow the reverse trend as compared to that of miR-490-5p; it elevated from days 7 to 28 and then showed a mild decline by day 35 (Figure 4E). To further study the effect of PITPNM1 on chondrogenesis, siPITPNM1 were transfected into hADSCs, which were subsequently induced to differentiate into chondrogenic microspheres for 21 days. Alcian Blue staining revealed that PITPNM1 knockdown suppressed the chondrogenesis of hADSCs (Figure 4F), which was similar to the effect of miR-490-5p overexpression. PITPNM1 was up-regulated in the cartilages from three patients with OA compared to those from three paired controls as determined by qRT-PCR (Figure 4G). Moreover, a down-regulated expression pattern of PITPNM1 was found in PHCs, the *in vitro* OA model (Figure 4H).

Interestingly, in stark contrast with miR-490-5p knockdown, the knockdown of PITPNM1 led to down- and up-regulation of ECM anabolism and catabolism markers, respectively, as determined by qRT-PCR (Figure 4I), WB (Figure 4J), and IF (Figure 4K). These findings strongly indicated that miR-490-5p might play an integral role in hADSC chondrogenesis and chondrocyte phenotypes by targeting PITPNM1. Further, dual-luciferase reporter assay was performed to determine whether the 3′-UTR of PITPNM1 contains an miR-490-5p interaction sequence. As indicated in Figure 4L, the 3′-UTR of PITPNM1 was found to contain putative binding sites for miR-490-5p, and the sequence ATCCATG was subsequently mutated into GCAAGCT. The luciferase activity of WT PITPNM1 was found to vary substantially when miR-490-5p was knocked down or overexpressed. In contrast, after PITPNM1 was mutated (MUT), its luciferase activity did not show a marked change when miR-490-5p was knocked down or overexpressed (Figure 4M). Taken together, the above findings signified that miR-490-5p targeted PITPNM1 by binding to the 3′-UTR of PITPNM1.

### miR-490-5p Activated the PI3K/AKT Signal Pathway in PHCs

**FIGURE 5:**
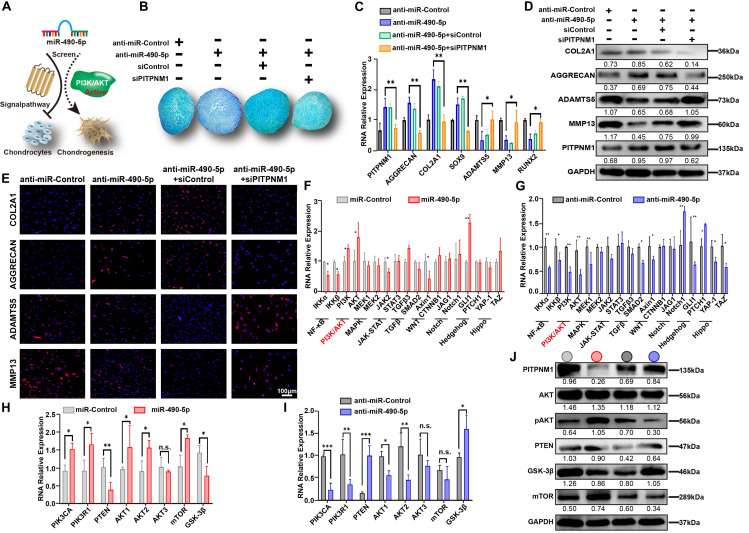
miR-490-5p activates the PI3K/AKT signal pathway in PHCs. **(A)** Schematic diagram illustrating the negative role played by miR-490-5p in PHCs and chondrogenesis by activating the PI3K/AKT signal pathway. **(B)** After transfection with anti-miR-490-5p alone or following PITPNM1 knockdown and control, hADSCs were induced to undergo chondrogenesis in 3D cultures for 14 days; Alcian Blue was used to stain the pathological section of microspheres. After PHCs were transfected with anti-miR-490-5p alone or following PITPNM1 knockdown, the expression levels of COL2A1, AGGRECAN, SOX9, MMP13, ADAMTS5, and RUNX2 were examined by **(C)** qRT-PCR, **(D)** Western blot, and **(E)** immunofluorescence. Gene expression of two major components of each principal signal pathway was evaluated by qRT-PCR after PHCs were transfected with **(F)** miR-490-5p or **(G)** anti-miR-490-5p. Gene expression of major components of the PI3K/AKT signal pathway was evaluated by qRT-PCR after PHCs were transfected with **(H)** miR-490-5p or **(I)** anti-miR-490-5p. **(J)** The protein levels of PITPNM1, AKT, pAKT, PTEN, GSK-3β, and mTOR in the indicated cells were determined by Western blot, the light gray circle denoted miR-Control, the red circle denoted miR-490-5p, the dark gray circle denoted anti-miR-Control and the blue circle denoted anti-miR-490-5p. *p < 0.05, **p < 0.01, n.s.: no significance.

To better understand the specific roles of miR-490-5p and PITPNM1 in hADSC chondrogenesis and chondrocytes, a rescue experiment was designed. miR-490-5p knockdown up-regulated PITPNM1 expression. Notably, compared with the anti-miR-control, the anti-miR-490-5p strengthened the chondrogenic ability of hADSCs (Figure 5B), as well as remarkably elevated the expression levels of ECM anabolism markers, while reducing that of ECM catabolism markers in PHCs (Figures 5C–E). However, co-transfection with siPITPNM1 restored the above effects caused by miR-490-5p knockdown. Collectively, these results suggested that miR-490-5p might negatively affect chondrogenesis and chondrocytes by targeting PITPNM1.

To identify a potential signaling pathway affected by miR-490-5p, the expression patterns of two vital molecules involved in each of the nine principal signaling pathways (NF-κB, PI3K/AKT, MAPK, JAK-STAT, TGFβ, WNT, Notch, Hedgehog, and Hippo signal pathways) were examined in PHCs transfected with either miR-490-5p mimic or inhibitor (Figure 5A). As shown in Figures 5F,G, the expression levels of PI3K and AKT demonstrated a similar mirror effect between miR-490-5p mimic and inhibitor, which suggested that miR-490-5p might affect PI3K/AKT signaling pathway. Then, the influence of miR-490-5p on these key molecules of PI3K/AKT signaling pathway was evaluated by RT-PCR (Figures 5H,I) and WB (Figure 5J); the expression of pAKT was significantly elevated while that of PTEN and GSK-3β was remarkably reduced following miR-490-5p overexpression. Moreover, miR-490-5p knockdown reversed the expression patterns of these molecules. However, as the upstream molecule of PI3K/AKT signal pathway, the expression of mTOR was not significantly affected by miR-490-5p. These findings revealed that miR-490-5p overexpression could activate PI3K/AKT/GSK-3β signaling pathway. Our data suggested a positive correlation between miR-490-5p and the PI3K/AKT signaling pathway; miR-490-5p modulated hADSC chondrogenesis and chondrocytes via the PI3K/AKT/GSK-3β/PITPNM1 axis.

### Inhibition of miR-490-5p Alleviated OA in the DMM Model

**FIGURE 6:**
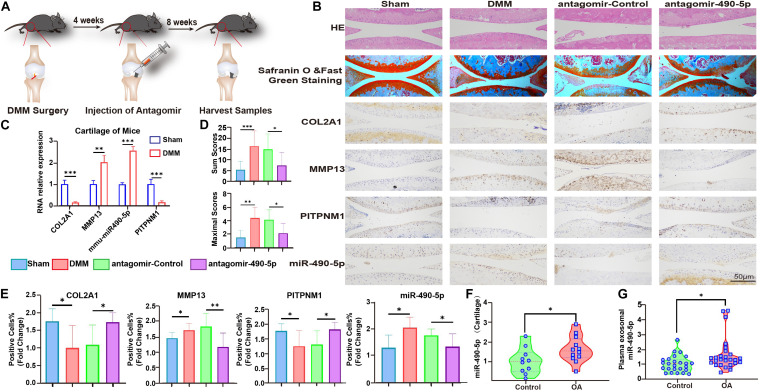
Mmu-anti-miR-490-5p remedied the effect of damaged cartilage in DMM OA mice. **(A)** Schematic diagram illustrating the procedure of *in vivo* experiment. **(B)** Safranin O and Fast Green Staining, *in situ* hybridization of miR-490-5p, and immunohistochemistry analysis of COL2A1, MMP13, and PITPNM1 are shown in sections of knee joints from mice. **(C)** qRT-PCR revealed the expression pattern of miR-490-5p and PITPNM1 in cartilage from mice subjected to sham (Control) or DMM surgery. **(D)** OARSI scores of the knee joints of DMM mice; the upper bar chart shows the sum scores of four cohorts, and the lower bar chart shows the maximal scores of four cohorts. **(E)** Fold change of positive cell rates of COL2A1, MMP13, PITPNM1, and miR-490-5p. **(F)** Expression pattern of miR-490-5p in 22 cartilage specimens from healthy population (Control) and patients with osteoarthritis (OA), as determined by qRT-PCR; GAPDH was used as the internal reference. **(G)** qRT-PCR revealed the expression pattern of miR-490-5p in 51 plasma samples from healthy population (Control) and patients with osteoarthritis (OA), as determined by qRT-PCR; Ce-mir-39 was used as the internal reference. ^∗^*p* < 0.05, ^∗∗^*p* < 0.01, ^∗∗∗^*p* < 0.001, n.s.: no significance.

To gain further insight into the impact of miR-490-5p *in vivo*, antagomir-490-5p was administered to the DMM mice model. Knee joints from antagomir-490-5p- and antagomir-control-treated mice were harvested and analyzed histologically (Figure 6A). Staining with hematoxylin–eosin (HE) and Safranin O and Fast Green revealed that the Osteoarthritis Research Society International (OARSI) scores of the knee joints in the antagomir-490-5p group were lower than those in the control group. Moreover, IHC showed that antagomir-490-5p decreased the expression level of MMP13 and increased that of COL2A1 *in vivo*, while mirror expression patterns for PITPNM1 and miR-490-5p were observed using qRT-PCR, IHC, and hybridization *in situ* (Figures 6B–E). Taken together, these observations suggested that miR-490-5p knockdown alleviated OA in the DMM model.

### Expression Levels of miR-490-5p in Clinical Cartilage and Plasma Samples

Finally, to evaluate the clinical relevance of miR-490-5p, the expression levels of miR-490-5p were determined in clinical cartilage specimens and plasma samples obtained from patients with OA and corresponding healthy controls. qRT-PCR revealed that miR-490-5p expression was markedly elevated in cartilage specimens from 12 patients with OA compared to that in healthy controls (Figure 6F). In addition, plasma exosomal miR-490-5p expression was significantly higher in 26 patients with OA compared to that in healthy controls (Figure 6G).

**FIGURE 7:**
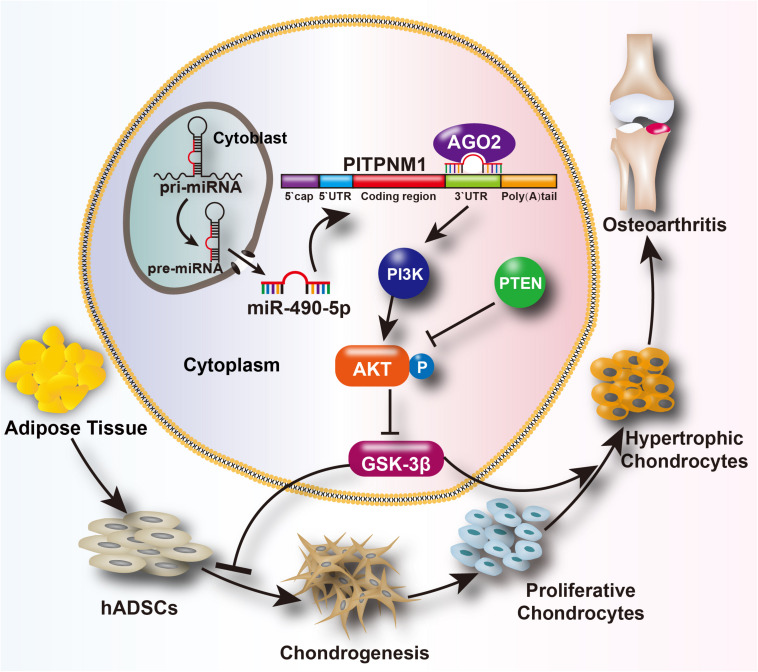
Schematic depiction of the mechanism of the miR-490-5p/PI3K/AKT/PITPNM1 axis on chondrogenesis and PHC degeneration. We hypothesizes that miR-490-5p can attenuate human adipose derived stem cell (hADSC) chondrogenesis and accelerate cartilage degradation. These effects can be reached probably through the activation of PI3K/AKT signal pathway by targeting PITPNM1.

In summary, inhibition of miR-490-5p enhanced hADSC chondrogenesis and prevented cartilage degradation. Moreover, miR-490-5p regulated the chondrogenic potential of hADSCs and phenotype of PHCs by targeting the PITPNM1/PI3K/AKT axis. The findings of the present study and their potential mechanisms are summarized in Figure 7.

## Discussion

In the last decade, miRNAs have captured widespread interest due to their critical regulatory effect at the post-transcriptional level, which affects cell function and phenotype in several diseases including cancer and degenerative diseases. miRNAs are considered to play vital roles in maintaining both chondrogenic potential and cartilage homeostasis (Swingler et al., 2019). Our previous researches have demonstrated that miR-193b (Hou et al., 2015), miR-455-3p (Mao et al., 2019), miR-92a (Mao et al., 2018), and miR-320c (Sun et al., 2019) are closely involved in modulating chondrogenesis and chondrocyte degeneration. Previous studies have revealed that miR-490-5p functions as a tumor suppressor in bladder cancer (Wu et al., 2019), hepatocellular carcinoma (Fang et al., 2018), renal cell carcinoma (Chen et al., 2016), and childhood neuroblastoma (Wang et al., 2020). Moreover, miR-490-5p is reported to facilitate the progression of irritable bowel syndrome (Ren et al., 2017). Yang et al. (2015) reported that miR-490-5p was gradually down-regulated during hADSC chondrogenic differentiation, and its overexpression decreased the expression of chondrogenic markers, such as COL2A1, COL10A1, and AGGRECAN. The abovementioned role of miR-490-5p in hADSC chondrogenesis is consistent with our findings; however, the definite effect and mechanism of miR-490-5p on chondrogenesis and chondrocyte phenotype remain largely unknown. In our study, we demonstrated for the first time that miR-490-5p inhibited chondrogenesis and facilitated cartilage phenotype degradation by sponging PITPNM1. Moreover, we found that miR-490-5p plays a negative role in chondrogenic differentiation and cartilage phenotype maintenance by activating the PI3K/AKT signal pathway. We consistently found miR-490-5p to be down-regulated in the differentiated microspheres. Its knockdown promoted the chondrogenic potential of hADSCs and strengthened the phenotype of PHCs *in vitro* and *in vivo*. These findings suggested miR-490-5p to be an underlying regulator of OA. Notably, our study found that overexpression of miR-490-5p could result in the up-regulation of RUNX2 and down-regulation of SOX9, suggesting that miR-490-5p overexpression promoted hADSC transition from chondrogenesis to osteogenesis. That is, miR-490-5p influenced the direction of hADSC differentiation.

We further identified PITPNM1 as the target of miR-490-5p using prediction database and subsequently verified this through several experiments. Previous studies indicated that PITPNM1 could promote breast cancer metastasis by intensifying epithelial–mesenchymal transition (Keinan et al., 2014) and regulates phosphoinositide signaling (Kim et al., 2013). However, to the best of our knowledge, the effect of PITPNM1 on OA has not been fully elucidated. Our study demonstrated that PITPNM1 has an opposite effect to that of miR-490-5p on chondrogenic potential and phenotype of chondrocytes. The effects of miR-490-5p overexpression were similar to that of PITPNM1 knockdown in OA. We subsequently identified that miR-490-5p silenced the expression of PITPNM1 by binding to its 3′-UTR, which could facilitate its degradation or suppress the translation of PITPNM1. Thus, we identified PITPNM1 as the potential target of miR-490-5p, providing a novel therapeutic target for OA.

To obtain a translational perspective, we explored the possible signaling pathways regulating the effects of OA induced by miR-490-5p and PITPNM1 by performing loss- and gain-of-function experiments and rescue assay. Out of the nine mainstream pathways that we screened, the PI3K/AKT signaling pathway was the most affected by miR-490-5p. We further validated the regulatory effect of the miR-490-5p/PITPNM1/PI3K/AKT axis on chondrogenic potential and chondrocytes. Similar to our findings, previous studies have also indicated that both miR-490-5p and PITPNM1 are closely related to the PI3K/AKT signaling pathway (Kim et al., 2013; Keinan et al., 2014; Chen et al., 2016). The PI3K/AKT signaling pathway, an intracellular signal transduction pathway, is largely involved in metabolism, proliferation, cell survival, growth, and angiogenesis (Hemmings and Restuccia, 2012). Moreover, recent evidence suggests that the PI3K/AKT/mTOR signaling pathway is indispensable for not only normal metabolism of joint tissues but also affecting the progression of OA (Sun et al., 2020). Thus, our study suggests that the miR-490-5p/PITPNM1/PI3K/AKT axis is a potential feasible target to limit and repair cartilage injury by facilitating hADSC chondrogenesis.

Cell therapy of hADSCs is one of the attractive therapeutic platforms for therapeutic strategy of a variety of diseases such as OA. Our data reveal for the first time the key role and potential mechanism of miR-490-5p in hADSC chondrogenesis. Given that, hADSCs have abundant multilineage potential and accessibility, such as infrapatellar fat pad or liposuction. Therefore, our findings might provide an effective targeted intervention for OA at early stage by using a stem cell therapeutic strategy. However, our study is limited in basic research; clinical translational verification and research are further needed. Moreover, in our study, the average age was significantly different between the OA and healthy participants (*p* < 0.05). However, Hunter and Bierma-Zeinstra (2019) reported that 26.6% of people aged 45 years and older suffer from OA, which suggested that the age of 45 years and older might not be a potential factor for the data from OA and participants because of the heterogeneity of the disease. To minimize the discrepancy, older healthy participants were preferentially enrolled in our study, but older people with total healthy joints are few.

## Conclusion

We found that miR-490-5p was down-regulated in hADSC chondrogenesis. Inhibition of miR-490-5p promoted the chondrogenic potential of hADSCs and protected the phenotype of chondrocytes by targeting the PITPNM1/PI3K/AKT axis. Our study identified that the miR-490-5p/PITPNM1/PI3K/AKT axis was a critical regulator of chondrogenic potential of hADSCs and chondrocyte phenotype. As it is relatively feasible to obtain adipose tissue in clinic, our findings provided novel insights into the potential strategy for OA treatment based on miR-490-5p and hADSCs. Thus, our findings provided a might provide a potential and novel stem cell therapeutic target for the treatment of OA.

## Data Availability Statement

The datasets presented in this study can be found in Supplementary Material.

## Ethics Statement

This study was approved by the First Affiliated Hospital of Sun Yat-sen University Clinical Research Ethics Committee ([2013] C-110). All participants in this study provided signed informed consent.

## Author Contributions

HL conceived, designed, and performed research, and wrote the manuscript. XZ collected clinical specimens, analyzed and interpreted the data, performed vivo experiments, and prepared figures. XW conceived, designed, and performed experiments. AZ and RL performed *in vitro* experiments. SH and GM performed *in vivo* experiments. WL supervised the study and revised the manuscript. ZZ conceived, designed, and supervised the study, and revised the manuscript. All authors contributed to the article and approved the submitted version.

## Conflict of Interest

The authors declare that the research was conducted in the absence of any commercial or financial relationships that could be construed as a potential conflict of interest.
